# Subclinical dysfunction of remote myocardium is related to high NT-proBNP and affects global contractility at follow-up, independently of infarct area

**DOI:** 10.3389/fcvm.2022.997821

**Published:** 2022-12-19

**Authors:** Giovanni Diana, Gabriella Locorotondo, Laura Manfredonia, Francesca Graziani, Antonella Lombardo, Gaetano Antonio Lanza, Daniela Pedicino, Giovanna Liuzzo, Massimo Massetti, Filippo Crea

**Affiliations:** ^1^Department of Cardiovascular Sciences, Fondazione Policlinico Universitario A. Gemelli Istituto di Ricovero e Cura a Carattere Scientifico, Rome, Italy; ^2^Department of Cardiovascular Sciences, University of the Sacred Heart, Rome, Italy

**Keywords:** remote myocardium, ischemic myocardium, border myocardium, subtle contractile dysfunction, NT-proBNP, longitudinal strain, multi-vessel coronary artery disease

## Abstract

**Background:**

In ST-segment elevation myocardial infarction (STEMI), predictors of subclinical dysfunction of remote myocardium are unknown. We prospectively aimed at identifying clinical and biochemical correlates of remote subclinical dysfunction and its impact on left ventricular ejection fraction (LVEF).

**Methods:**

One-hundred thirty-three patients (63.9 ± 12.1 years, 68% male) with first successfully treated (54% anterior, 46% non-anterior, *p* = 0.19) STEMI underwent echocardiography at 5 ± 2 days after onset and at 8 ± 2-month follow-up, and were compared to 13 age and sex-matched (63.3 ± 11.4) healthy controls. All 16 left ventricular (LV) segments were grouped into ischemic, border, and remote myocardium: mean value of longitudinal strain (LS) within grouped segments were expressed as iLS, bLS, rLS, respectively. LV end-diastolic (EDV), end-systolic (ESV) volumes indexed for body surface area (EDVi, ESVi, respectively), LVEF and global LS (GLS) were determined. Creatinine, glomerular filtration rate, admission level of NT-pro-brain-natriuretic peptide (NT-proBNP) and troponin peaks were considered for the analysis.

**Results:**

At baseline, rLS (15.5 ± 4.4) was better than iLS (12.9 ± 4.8, *p* < 0.001), but lower than that in controls (19.1 ± 2.7, *p* < 0.001) and similar to bLS (15 ± 5.4, *p* = ns), and did not differ between patients with single or multivessel coronary artery disease (CAD). At multivariate regression analysis, only admission NT-proBNP levels but not peak Tn levels independently predicted rLS (β = −0.58, *p* = 0.001), as well as iLS (β = −0.52, *p* = 0.001). Both at baseline and at follow-up, rLS correlated to LVEF similarly to iLS and bLS (*p* < 0.001 for all). Median value of rLS at baseline was 15%: compared to patients with rLS ≥ 15% at baseline, patients with rLS < 15% showed lower LVEF (52.3 ± 9.4 vs. 58.6 ± 7.6, *p* < 0.001) and GLS (16.3 ± 3.9 vs. 19.9 ± 3.2), and higher EDVi (62.3 ± 19.9 vs. 54 ± 12, *p* = 0.009) and ESVi (30.6 ± 15.5 vs. 22.3 ± 7.6, *p* < 0.001) at follow-up.

**Conclusion:**

In optimally treated STEMI, dysfunction of remote myocardium assessed by LS: (1) is predicted by elevated NT-proBNP; (2) could be independent of CAD extent and infarct size; (3) is associated to worse LV morphological and functional indexes at follow-up.

## Introduction

ST-Segment Elevation Myocardial Infarction (STEMI) is the most acute manifestation of coronary artery disease (CAD). Although current therapies have significantly decreased death for STEMI in the past 3 decades (1), long-term mortality remains high (2, 3). Recent evidence by cardiac magnetic resonance (CMR) suggests that infarct size and microvascular obstruction are not the only predictors of adverse outcome (4): structural changes, such as interstitial fibrosis, occurring outside infarcted area as maladaptive response to the ischemic injury (5), are associated with persistent left ventricular (LV) contractile dysfunction at follow-up (6) and predict long-term LV remodeling, independently of infarct size and microvascular obstruction (7).

Characterization of myocardium inside and outside infarcted area can be made only by using sophisticated imaging tools, since assessment of LV ejection fraction (LVEF) or wall motion abnormalities is poorly sensitive in catching subtle functional changes within viable myocardium (8). Based on the automatic analysis of myocardial deformation, strain imaging is capable to identify subtle global and regional contractile dysfunction (9). In the setting of STEMI, strain has been used by echocardiography and CMR mainly to differentiate between transmural and non-transmural infarctions and to predict infarct size (10) and death or HF in addition to LVEF (11). Interestingly, an impairment of strain parameters has been demonstrated beyond ischemic area in patients with single-vessel CAD (12). Longitudinal strain (LS) has been found to considerably overlap between infarcted and non-infarcted myocardium (8, 13), and, even in segments visually considered normokinetic, regional LS is significantly reduced compared with healthy controls (14).

Biochemical predictors of subtle contractile dysfunction of remote myocardium, as well as its functional impact in comparison with infarct and borderline myocardium, have not been investigated before in large cohorts of STEMI patients. In this prospective single center study, we aimed at: (1) detecting predictors of LS in remote myocardium, as compared to infarct and borderline myocardium, by echocardiographic LS, during the sub-acute phase of first successfully treated STEMI; (2) verifying its impact on LVEF; (3) assessing echocardiographic data at 6-month follow-up, stratified by value of baseline LS in remote myocardium.

## Materials and methods

### Patient population

A consecutive series of patients admitted to our Intensive Cardiac Care Unit for a first STEMI, from February 2018 to May 2021, was screened. The diagnosis of STEMI was based on the presence of typical chest pain or equivalents, lasting at least 20 min, associated with the persistence of the ST-segment elevation (0.1 mV in 2 or more peripheral leads or 0.2 mV in 2 or more precordial contiguous leads) and elevation of cardiac troponin (Tn) and creatine-kinase MB fraction (CK-MB) with at least a value above the 99th percentile of the upper reference limit (15). Patients were treated by primary (within 12 h of symptom onset) or rescue percutaneous coronary intervention (PCI), with achievement of successful recanalization of the culprit coronary artery, denote by a Thrombolysis in Myocardial Infarction (TIMI) 3 flow grade. Patients with multivessel CAD were subsequently treated by PCI, 2–4 days later and were enrolled after completion of coronary revascularization. In accordance with current guidelines (15), all patients were treated by dual antiplatelet therapy, beta-blockers, ACE-inhibitors and statins throughout the study, and none had to prematurely stop therapy until follow-up. In order to avoid any confounding impact of residual significant stenosis on culprit and non-culprit coronary arteries, as well as any periprocedural necrosis related to previous revascularization, on strain values (16, 17), we elected to exclude patients with achievement of TIMI flow grade ≤ 2 on culprit and non-culprit coronary arteries. Other exclusion criteria were: (1) a concomitant moderate/severe mitral and/or aortic valve disease; (2) cardiogenic shock, electrical/hemodynamic instability, endotracheal intubation; (3) a history of concomitant cardiomyopathy; (4) previous percutaneous or surgical myocardial revascularization; (5) residual significant coronary artery stenosis, that could not be revascularized. A group of 13 age and sex matched subjects without previous overt cardiovascular disease, undergoing echocardiography at our Outpatient Cardiology Clinic as screening tool, served as controls. The study protocol was conducted in agreement with the Declaration of Helsinki for Humans Rights. Each enrolled patient gave written informed consent to participate in the study.

### Biochemical assessment

Blood samples for the dosage of high sensitivity Tn, NT-pro-brain-natriuretic peptide (NT-proBNP) and creatinine serum levels were obtained upon arrival of the patient in the emergency room. Subsequently, dosage of Tn was repeated after PCI, at 6 and 12 h and then daily until normalization of values or discharge. Although it is weak to assess the infarct size, peak values of Tn was considered for the analysis, while value of NT-proBNP and creatinine at admission were taken into account, in order to avoid effect of treatment on serum level of biomarkers.

### Echocardiographic assessment

Transthoracic echocardiography was performed by using last generation ultrasound machines (Epiq 7C and CVX, Philips, Milan, Italy) equipped with a two-dimensional X3 probe, both at baseline (i.e., prior to discharge) and at 6-month follow-up. LV end-diastolic (EDV) and end-systolic (ESV) volumes were obtained from 4- and 2-chamber apical views using the modified Simpson’s biplane method, and were indexed for body surface area (EDVi and ESVi, respectively). LVEF was calculated as [(EDV-ESV)/EDV] × 100.

For regional contractile function assessment, a 16-segment myocardial model was used, according to the recommendations of the American Heart Association (18). All myocardial segments were grouped into infarcted, border and remote myocardial zones, according to a standardized coronary artery territory scheme (18, 19) by an investigator (GL) blinded to echocardiographic data, taking into account results of coronary angiography: particularly, LV segments were divided basing on the culprit coronary artery (with its distribution territory) and the relative dominance of coronary circulation seen at coronary angiography. Thus, the ischemic myocardium was labeled as myocardial territory supplied by culprit coronary artery, considering dominance of coronary circulation at angiography, while remote myocardium was identified as the myocardium opposite of 180° to the ischemic region. Myocardial segments between ischemic and remote myocardium were considered as border zone.

Analysis of LS was automatically performed off-line, by tracing endocardial border in apical 4, 3, and 2-chamber views, using Autostrain package software (Tomtec, Philips, Italy). Global LS (GLS) was obtained as mean value of the three views and LS values in each myocardial segment were considered. The mean value of LS within ischemic, border, and remote zones was taken into account for the analysis and expressed as iLS, bLS, and rLS, respectively. GLS, iLS, bLS, and rLS were reported as absolute values. Patients with suboptimal images were excluded from the analysis.

### Statistical analysis

Statistical analysis was performed using SPSS 21.0 statistical software (SPSS Inc., Florence, Italy). After checking for normality distribution by Kolmogorov-Smirnov test, continuous variables were expressed as mean ± standard deviation (SD) if parametric and as logarithmic transformation if non-parametric. Categorical variables were expressed as percentage. Differences were assessed by Student *t*-test or one-way ANOVA for continuous variables or by Chi-square test for categorical variables. Multiple comparisons were corrected by Bonferroni method. Correlations were tested by Pearson test and compared by z-statistics. Univariate and multivariate linear regression analyses were performed: predictors with *p* ≤ 0.10 at univariate analysis entered multivariate analysis, which was adjusted for age, hypertension, diabetes, dyslipidemia, smoking, and presence of pre-infarctual angina. Within-group differences between baseline and follow-up were analyzed by two-way ANOVA for repeated measures. Finally, all patients were stratified into 2 groups, basing on median value of rLS at baseline: differences between the two groups in clinical, biological and echocardiographic data were assessed both at baseline and at follow-up. A *p*-value < 0.05 was considered as statistical significance for all analyses.

## Results

### General characteristics of study population

**FIGURE 1:**
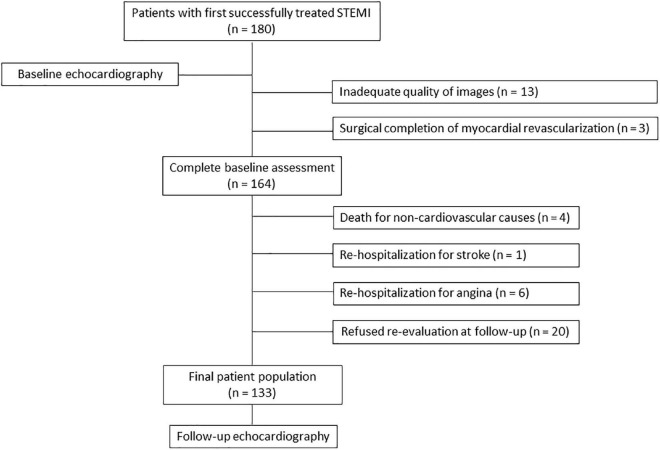
Study flow-chart. STEMI, ST-segment elevation myocardial infarction.

Out of 180 patients enrolled at baseline, 47 patients were subsequently excluded (Figure 1). Thus, 133 patients (63.9 ± 12.1 years, 85% male) formed our patient population. Clinical and echocardiographic characteristics of patients are summarized in Table 1, together with clinical findings of healthy control subjects. Most patients (*n* = 77, 58%) had single-vessel CAD, while 42 (32%) had two-vessel and 14 (10%) three-vessel CAD (overall *p* = 0.01). Seventy-two (54%) patients presented with anterior STEMI, while 61 (46%) patients with non-anterior STEMI (*p* = 0.19).

**TABLE 1:** Clinical and echocardiographic data in patients and in controls at baseline.

	Patient population (*n* = 133)	Controls (*n* = 13)	*p*
Age, yrs, mean ± SD	63.9 ± 12.1	63.3 ± 11.4	0.78
Male, *n* (%)	90 (68)	6 (46)	0.11
Hypertension, *n* (%)	73 (55)	9 (69)	0.33
Diabetes, *n* (%)	30 (23)	3 (23)	1
Dyslipidemia, *n* (%)	54 (41)	6 (46)	0.73
Smoking, *n* (%)	61 (46)	6 (46)	1
Pre-infarctual angina, *n* (%)	39 (29)	/	
Anterior STEMI, *n* (%)	72 (54)	/	
Door-to-balloon time, hrs, mean ± SD	6.7 ± 5.1	/	
Creatinine, mg/dL, mean ± SD	1 ± 0.2	0.76 ± 0.23	0.0002
GFR, ml/min, mean ± SD	78.4 ± 22.8	105.2 ± 52.8	0.0029
NT-proBNP, pg/L, median (interquartile range)	1646 (763 − 4113.50)	/	
Troponine, ng/L, median (interquartile range)	378.76 (103.68 − 84324)	/	
EDVi, ml/m^2^, mean ± SD	51.3 ± 12.5	26.9 ± 3.2	<0.001
ESVi, ml/m^2^, mean ± SD	26.3 ± 10.2	13.1 ± 11.2	<0.001
LVEF, %, mean ± SD	49.4 ± 10.4	67.7 ± 4.5	<0.001
GLS, %, mean ± SD	14.8 ± 4.9	21.2 ± 1.4	<0.001
iLS, %, mean ± SD	12.9 ± 4.8	19.1 ± 2.1	0.002
bLS, %, mean ± SD	15 ± 5.4	18.9 ± 2.8	0.013
rLS, %, mean ± SD	15.5 ± 4.4	19.1 ± 2.7	0.005

bLS, border longitudinal strain; bWMS, border wall motion score; EDVi, end-diastolic volume indexed for body surface area; EF, ejection fraction; ESVi, end-systolic volume indexed for body surface area; GFR, glomerular filtration rate; GLS, global longitudinal strain; iLS, ischemic longitudinal strain; iWMS, ischemic wall motion score; NT-proBNP, natriuretic peptide; rLS, remote longitudinal strain; rWMS, remote wall motion score; SD, standard deviation; STEMI, ST segment elevation myocardial infarction.

Baseline echocardiography was performed at 5 ± 2 days from STEMI onset. LS significantly differed among the three myocardial zones (overall *p* < 0.001), with rLS being higher than iLS (*p* < 0.001), but similar to bLS (*p* = 0.99) and lower than controls (*p* < 0.001). ILS also differed from bLS (*p* = 0.003) (Table 1).

### Clinical and biochemical correlates of LS values at baseline

**FIGURE 2:**
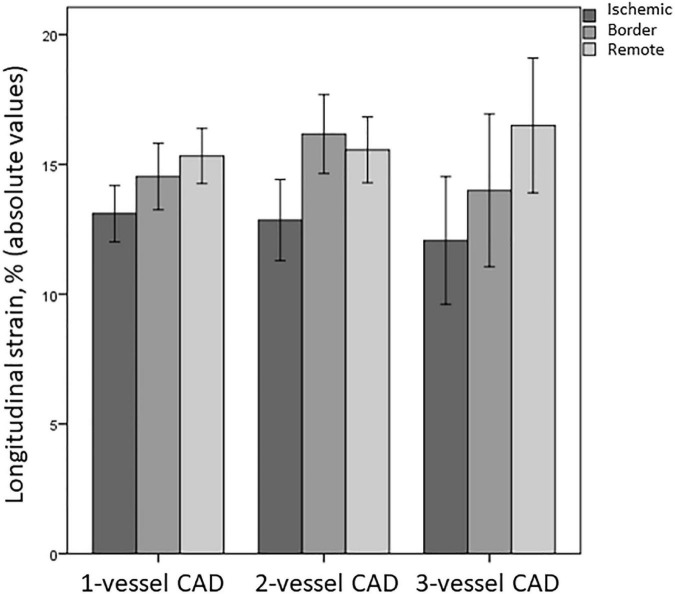
Differences in LS of ischemic, border, and remote myocardium between patients with single-vessel, two-vessel, and three-vessel CAD. CAD, coronary artery disease.

Multi-vessel CAD did not affect LS more than single-vessel CAD: nor rLS (*p* = 0.66), neither iLS (*p* = 0.76), or bLS (*p* = 0.22) differed between patients with single-vessel and multi-vessel CAD (Figure 2). Similarly, no difference was found in GLS and LVEF between patients with single-, two-, and three vessel CAD (14.6 ± 5 vs. 15.5 ± 4.4 vs. 14.4 ± 5, respectively, for GLS, overall *p* = 0.55; 49.4 ± 10.6 vs. 50 ± 10.3 vs. 47.4 ± 10, respectively, for LVEF, overall *p* = 0.72).

Univariate and multivariate analyses were performed separately for rLS, iLS, and bLS. Results of univariate analysis are reported in Table 2. After adjustment for age, cardiovascular risk factors and presence of pre-infarctual angina, only NT-proBNP values at admission independently predicted rLS (β = −0.58, 95% CI −5.43 to −1.68, *p* = 0.001), whereas iLS was predicted by NT-proBNP (β = −0.52, 95% CI −5.91 to −1.57, *p* = 0.001) and creatinine values (β = −0.34, 95% CI −9.51 to −0.52, *p* = 0.03), and bLS by non-anterior location of STEMI (β = −0.45 for anterior STEMI, 95% CI −8.00 to −1.42, *p* = 0.007).

**TABLE 2:** Results of univariate linear regression analysis for regional LS.

	iLS			bLS			rLS		
	Beta	95% CI	*p*	Beta	95% CI	*p*	Beta	95% CI	*p*
Anterior STEMI	-0.12	–2.76 to 0.51	0.18	-0.51	–7.86 to –4.85	<0.001	-0.48	–5.62 to –2.93	<0.001
Door-to-balloon time	-0.005	–0.15 to 0.14	0.96	0.05	–0.12 to 0.19	0.63	-0.06	–0.18 to 0.10	0.57
Number of CAD vessels	-0.057	–1.60 to 0.81	0.52	0.044	–1.02 to 1.71	0.62	0.07	–0.65 to 1.59	0.41
Creatinine	-0.29	–8.84 to –2.32	0.001	-0.14	–6.85 to 0.77	0.12	-0.25	–7.78 to –1.47	0.004
GFR	0.17	–0.002 to 0.072	0.064	0.09	–0.02 to 0.6	0.31	0.26	0.02–0.09	0.004
Troponin	-0.54	–2.13 to –1.20	<0.001	-0.37	–1.85 to –0.69	<0.001	-0.18	–1.04 to –0.001	0.05
NT-proBNP	-0.32	–4.25 to –0.78	0.005	-0.34	–5.29 to –1.15	0.003	-0.41	–4.58 to –1.42	<0.001

### rLS and ventricular function at baseline

**FIGURE 3:**
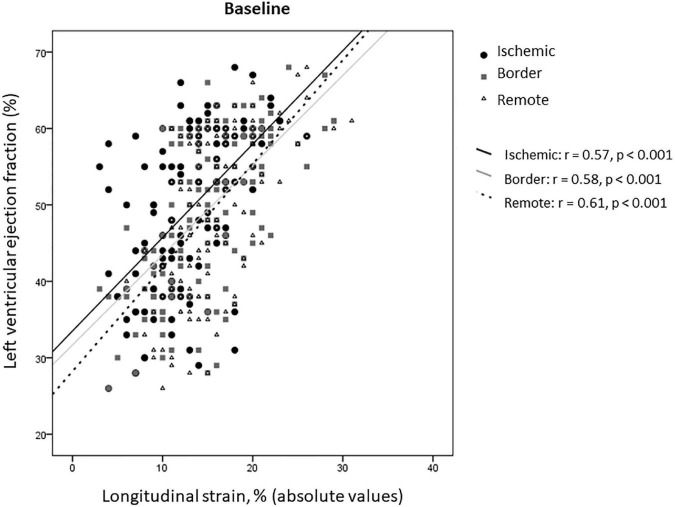
Correlations between LS of ischemic, border, and remote myocardium and LVEF at baseline.

In the overall study population, rLS correlated with LVEF at baseline, similarly to iLS and bLS (*z*-Test −0.166, *p* = 0.43 for comparison of correlation coefficients) (Figure 3).

Median value of rLS at baseline was 15%: according to such value, patients were stratified into group 1 (median rLS < 15%, *n* = 67) and group 2 (median rLS ≥ 15%, *n* = 66). Patients with baseline rLS < 15% more frequently had anterior STEMI and lower renal function, whereas showed only a trend to greater infarct size than patients with baseline rLS ≥ 15% (Table 3). Moreover, patients with baseline rLS < 15% showed greater LV volumes and lower LVEF, GLS, iLS, and bLS than patients with baseline rLS ≥ 15% (Table 3).

**TABLE 3:** Differences in baseline clinical, echocardiographic, and biological data between patients stratified by median values of rLS.

	Total population (*n* = 133)	rLS < 15% (*n* = 67)	rLS ≥ 15% (*n* = 66)	*p*
Age, yrs, mean ± SD	63.9 ± 12.1	63.3 ± 18	60.4 ± 11.1	0.12
Hypertension, *n* (%)	90 (68)	35 (52)	38 (57)	0.74
Diabetes, *n* (%)	73 (55)	16 (24)	14 (21)	0.60
Dyslipidemia, *n* (%)	30 (23)	26 (39)	28 (42)	0.84
Smoking, *n* (%)	54 (41)	27 (40)	34 (51)	0.28
Pre-infarctual angina, *n* (%)	61 (46)	20 (30)	19 (29)	0.70
Anterior STEMI, *n* (%)	39 (29)	51 (76)	21 (32)	<0.001
Number of diseased coronary arteries, mean ± SD	72 (54)	1.5 ± 0.6	1.6 ± 0.7	0.18
Door-to-balloon time, hours, mean ± SD	6.7 ± 5.1	6.7 ± 5.1	6.6 ± 7.6	0.44
Creatinine, mg/dL, mean ± SD	1 ± 0.2	1 ± 0.2	0.9 ± 0.2	0.016
GFR, ml/min, mean ± SD	78.4 ± 22.8	78.4 ± 22.8	87.5 ± 20.1	0.028
NT-proBNP, pg/L, median (interquartile range)	1646 (763 − 4113.50)	2200 (1318 − 8586)	1158 (612 − 1718)	0.026
Troponine, ng/L, median (interquartile range)	378.76 (103.68 − 84324)	17485 (151.81 − 122081)	228.38 (85.21 − 68039)	0.055
EDVi, ml/m^2^, mean ± SD	51.3 ± 12.5	54.4 ± 12.8	48.3 ± 11.5	0.009
ESVi, ml/m^2^, mean ± SD	26.3 ± 10.2	30.4 ± 10.8	21.9 ± 7.4	<0.001
LVEF, %, mean ± SD	49.4 ± 10.4	44.9 ± 9.9	55 ± 8.2	<0.001
GLS, %, mean ± SD	14.8 ± 4.9	12.2 ± 3.4	18.1 ± 3.9	<0.001
iLS, %, mean ± SD	12.9 ± 4.8	11.3 ± 4.3	14.3 ± 4.7	0.002
bLS, %, mean ± SD	15 ± 5.4	11.5 ± 4	17.9 ± 4.5	<0.001
rLS, %, mean ± SD	15.5 ± 4.4	12.2 ± 2.1	18.9 ± 3.6	<0.001

bLS, border longitudinal strain; bWMS, border wall motion score; EDVi, end-diastolic volume indexed for body surphace area; EF, ejection fraction; ESVi, end-systolic volume indexed for body surphace area; GFR, glomerular filtration rate; GLS, global longitudinal strain; iLS, ischemic longitudinal strain; iWMS, ischemic wall motion score; NT-proBNP, natriuretic peptide; rLS, remote longitudinal strain; rWMS, remote wall motion score; SD, standard deviation; STEMI, ST segment elevation myocardial infarction.

### rLS at baseline and evolution at follow-up

**FIGURE 4:**
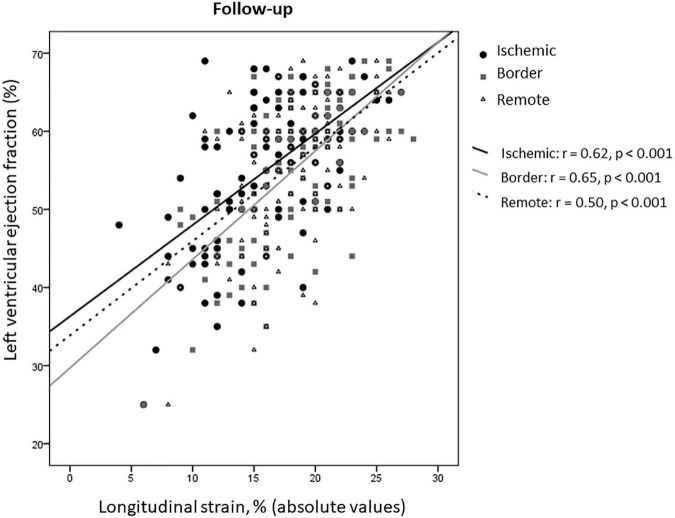
Correlations between LS of ischemic, border, and remote myocardium and LVEF at follow-up.

At 8 ± 2 months after STEMI, in the overall study population, both LVEF and GLS improved compared to baseline (55.5 ± 9 vs. 49.4 ± 10.4 and 18.1 ± 4 vs. 14.8 ± 4.9, respectively, *p* < 0.001 for both), as well as rLS (18 ± 3.7 vs. 15.5 ± 4.4 at baseline, *p* < 0.001), iLS (16.4 ± 4.7 vs. 12.6 ± 4.8 at baseline, *p* < 0.001), and bLS (18.6 ± 4.3 vs. 15 ± 5.4 at baseline, *p* < 0.001), while changes of EDVi (54.9 ± 26.4 vs. 51.3 ± 12.5 at baseline) and of ESVi (24.1 ± 14.2 vs. 26.3 ± 10.2 at baseline) were not significant (*p* = 0.43 and 0.13, respectively). At follow-up, rLS still correlated with LVEF similarly to iLS and bLS (z-statistic −0.59, *p* = 0.27) (Figure 4).

**FIGURE 5:**
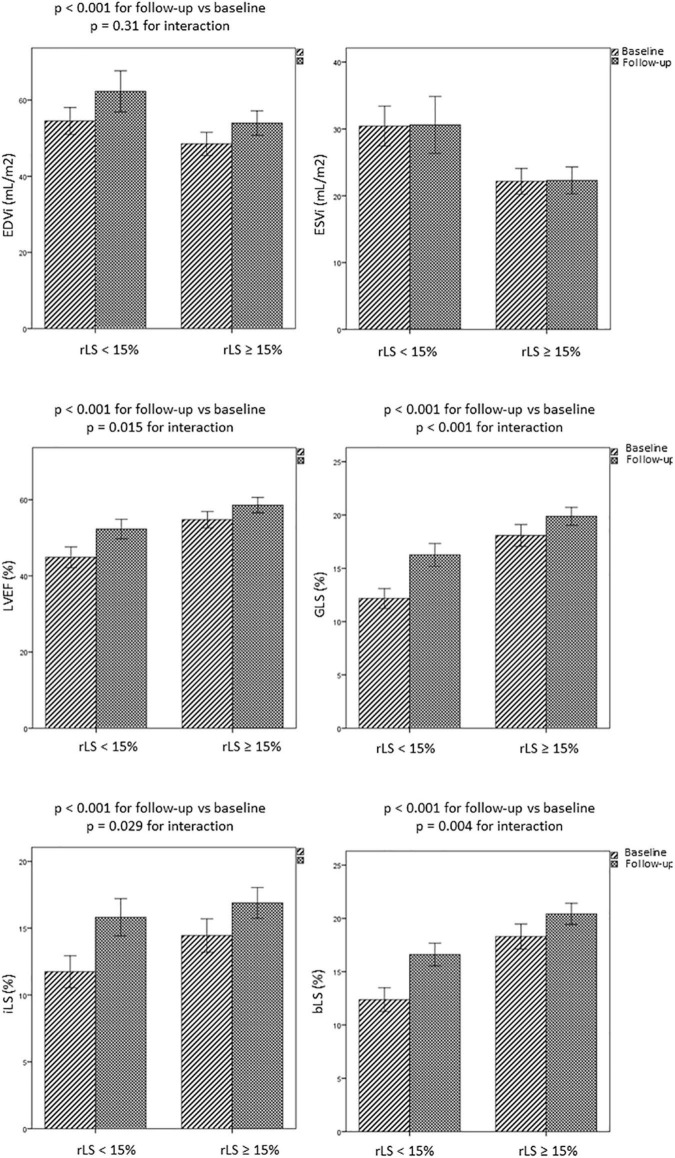
Evolution over time of LV volumes and regional and global LV function in patients with rLS < 15% and ≥ 15% at baseline. bLS and iLS, border and infarct longitudinal strain; EDVi and ESVi, end-diastolic and end-systolic volumes indexed for body surface area; GLS, global longitudinal strain; LVEF, left ventricular ejection fraction.

Interestingly, patients with baseline rLS < 15% showed greater improvement of regional and global LV contractility than patients with baseline rLS ≥ 15%, at the expense of greater increase of EDVi (Figure 5 and Table 4). However, at follow-up, patients with baseline rLS < 15% presented values of LVEF and GLS still lower, and LV volumes higher, than those of patients with baseline rLS ≥ 15%, despite similar iLS (Table 4).

**TABLE 4:** Differences in follow-up data between patients stratified by median values of rLS at baseline.

	Total population (*n* = 133)	rLS < 15% (*n* = 67)	rLS ≥ 15% (*n* = 66)	*p*
EDVi, ml/m^2^, mean ± SD	58 ± 16.8	62.3 ± 19.9	54 ± 12	0.009
ESVi, ml/m^2^, mean ± SD	26.3 ± 12.8	30.6 ± 15.5	22.3 ± 7.6	<0.001
LVEF, %, mean ± SD	55.5 ± 9	52.3 ± 9.4	58.6 ± 7.6	<0.001
GLS, %, mean ± SD	18.1 ± 4	16.3 ± 3.9	19.9 ± 3.2	<0.001
iLS, %, mean ± SD	16.4 ± 4.7	15.8 ± 5.1	16.9 ± 4.4	0.23
bLS, %, mean ± SD	18.6 ± 4.3	16.6 ± 3.9	20.4 ± 3.7	<0.001
rLS, %, mean ± SD	18 ± 3.7	16 ± 4.5	19.8 ± 2.9	<0.001

bLS, border longitudinal strain; bWMS, border wall motion score; EDVi, end-diastolic volume indexed for body surphace area; EF, ejection fraction; ESVi, end-systolic volume indexed for body surphace area; GFR, glomerular filtration rate; GLS, global longitudinal strain; iLS, ischemic longitudinal strain; iWMS, ischemic wall motion score; NT-proBNP, natriuretic peptide; rLS, remote longitudinal strain; rWMS, remote wall motion score; SD, standard deviation; STEMI, ST segment elevation myocardial infarction.

## Discussion

In a consecutive series of patients with first optimally treated STEMI and prospectively followed-up by echocardiography, we found that: (1) in the sub-acute phase of STEMI iLS, rLS, and bLS are independent of extent of CAD and are associated to LVEF; (2) NT-proBNP level at admission is an independent predictor of rLS; (3) despite an overall improvement of contractile function over time, patients with more impaired rLS at baseline show worse LV morphological and functional indexes also at follow-up. To the best of our knowledge, this is the first study attempting to characterize remote myocardium after ischemic injury by biochemical and imaging approach: our results suggest that impaired remote myocardial function as assessed by LS is associated to a worse LVEF at follow up which may eventually lead to heart failure (HF).

### Impairment of remote myocardium and relationship with CAD and LVEF

Our data about contractile dysfunction of remote myocardium in STEMI patients expand previous findings (8, 10, 13, 14), by highlighting that remote myocardium behaves similarly to border zone both during the sub-acute phase of STEMI and at follow-up. It may be due to the widespread inflammatory activation (20, 21) with maladaptive changes of the extracellular matrix (5), resulting in diffuse fibrosis (22, 23). The finding that rLS correlates with LVEF similarly to iLS and bLS confirms that structural and functional changes, occurring outside the infarcted area (24–27), might contribute to impair contractility (28). Regional LS can be affected independently of CAD extent, as well as perfusion abnormalities can be present outside infarct area in patients without multi-vessel CAD (29). In completely revascularized patients, it can be supposed that impairment of remote strain might relate to an abnormal response to ischemic injury, such as abnormal inflammatory response, rather than to non-culprit CAD itself. By this point of view, myocardium supplied by one or multivessel CAD may display similar impairment. Indeed, inflammatory response is independent of CAD extent (20). Discrepancies with previous data, showing that GLS was lower in multi-vessel than single-vessel CAD patients (30), probably can be justified by different completion of revascularization at the time of baseline echocardiography.

### Correlates of rLS impairment

Anterior STEMI particularly impairs rLS (30): indeed, our patients with rLS < 15% at baseline more frequently had anterior STEMI, although location of MI was not an independent predictor of rLS. Old age, high cardiovascular risk burden and reduced therapy intake increase the number of dysfunctional myocardial segments by strain (14), but they did not independently predict regional LS in our analysis. Similarly, although Tn peak can be particularly high in patients with depressed LVEF and impaired strain parameters (31), we did not find a direct relationship between LS and Tn levels, maybe because LS is less able to predict transmural necrosis than circumferential or radial strain (31). It is already known that, even in early stage, chronic kidney disease affects global LS in patients without previous cardiovascular events, due to diffuse myocardial fibrosis (32). No previous data particularly exist on the impairment of remote strain in patients with STEMI and chronic kidney disease, which further highlights the novelty of our research. On the other hand, we cannot exclude that our patients with renal impairment might have reduced strain values also prior to STEMI. In sharp contrast, we found that NT-proBNP value at admission independently predicted rLS. This is in accordance with data showing that NT-proBNP levels exhibit an association with GLS in acute myocardial infarction (33). Thus, the relationship of NT-proBNP with LS is stronger than that between Tn and LS, as demonstrated in a large spectrum of ischemic and non-ischemic cardiomyopathy and throughout the entire range of LVEF in HF (34, 35). We can suppose that NT-proBNP may have an adjunctive role in prediction myocardial dysfunction extending beyond infarct area: with the same extension of the infarct, different patients may present different functional impairment. Our findings underlie the concept that impaired LS is a marker of HF: the lower rLS, the higher NT-proBNP, the worse LVEF.

### Typifying at baseline, predicting follow-up: Clinical implications

LV strain predicts prognosis after AMI (36, 37): particularly, an elevated number of segments with abnormal strain is associated with all-cause mortality and death or hospitalization for HF (14). Thus, it has been suggested that low strain values outside the culprit lesion perfusion area might be risk stratifier better than GLS (38). However, no previous study has demonstrated that impairment of remote myocardium by LS may predict LV morphology and function at follow-up, thus conceivably explaining association with outcome. By taking into account the longitudinal component of myocardial strain, because of its well documented prognostic value (39), we found that patients with rLS < 15% at baseline had lower LVEF at follow-up, as compared to patients with rLS ≥ 15%, despite improvement of contractility over time. This is probably due to development of interstitial fibrosis in the remote myocardium, which is independently associated to LV dysfunction in patients with prior MI (6). Indeed, our patients with rLS < 15% at baseline had also reduced renal function, which notably affects LS, even in patients without previous cardiovascular events, due to diffuse myocardial fibrosis (32). The finding that rLS remained lower in patients with baseline rLS < 15% and impaired renal function, suggests that after resolution of inflammatory response a diffuse myocardial fibrosis might persists. The evidence of higher LV volumes at follow-up in patients with more depressed rLS, despite improvement of regional and global contractility, is also corroborated by the notion that there is dissociation between increase of EDVi at follow-up and functional improvement (40). For the first time, we can speculate that reduced rLS might be the mechanistic link between LV remodeling and function: remote myocardium contributes to LV remodeling, independently of infarcted myocardium.

In conclusion, for long time every effort in the setting of STEMI was made in the attempt to reduce the ischemic wavefront, as infarct size was considered one of the main determinants of adverse LV remodeling and outcome. Basing on our findings, however, patients with STEMI and high level of NT-proBNP since admission may have impairment of contractile function spreading beyond infarct area, regardless of CAD extent. Contribution of remote myocardium to global LV contractile function would explain why patients with similar extent of infarct area have different LVEF evolutions. If confirmed in further large cohorts of STEMI patients, the present findings may open the road toward treatments in the acute and sub-acute phase of STEMI, aiming not only to limit the ischemic wavefront, but also to preserve structural and functional integrity of myocardium surrounding necrosis, by decreasing the maladaptive response to the ischemic injury. Even patients with limited infarct size may need a careful follow-up in order to timely detect and prevent adverse remodeling. Future studies should be encouraged in order to test efficacy of a more aggressive pharmacological treatment, employing advanced HF therapy since the sub-acute phase of STEMI.

The most important limitation of our study is the lack of imaging data deriving from a tool able to precisely delineate infarct area, such as late gadolinium enhancement CMR. Current results should be confirmed with appropriate advanced myocardial imaging technique that are not available in the present study. However, although less precise than late gadolinium enhancement quantification, Tn peak has been previously validated as surrogate of infarct size (41). Differences in LS between infarct and non-infarcted segments may be greater than our ones when assessed early after myocardial reperfusion (10). Other than suggesting a time-dependent relationship, this evidence leads to exclude that the contractile function of the remote myocardium might be influenced by infarcted area throughout changes in regional mechanical loads (12). Unfortunately, laboratory data were missed at follow-up, because outpatient blood withdrawal was not initially planned at the time of follow-up echocardiography. Although relationship between NT-proBNP and GLS is widely documented in literature, and being rLS part of GLS, such similar correlation could be predictable, our purpose was just trying to unbundle remote from ischemic and border zones in order to differentially characterize it. In that, independence of remote strain by infarct size and CAD extent was not predictable and represents the core of our work. Probably, differences between our and previous studies relies on completeness of myocardial revascularization, which was not a goal in previous studies. Finally, although the only observational nature of our study may represent a limitation, pathophysiological mechanisms explaining our results may be supposed by previous findings. On the other hand, we can demonstrate how an automatic imaging tool, such as myocardial strain analysis, considered as artificial intelligence, may provide important insights, which on the contrary would be missed.

## Data availability statement

The original contributions presented in this study are included in the article/supplementary material, further inquiries can be directed to the corresponding author.

## Author contributions

GD collected the data and designed the analysis. GLo designed the analysis, performed the analysis, and wrote the manuscript. LM collected the data. FG, AL, DP, GLi, MM, and GAL reviewed the manuscript. FC reviewed and approved the manuscript. All authors contributed to the article and approved the submitted version.
